# Seroepidemiologic Survey of Crimean-Congo Hemorrhagic Fever Virus in Logging Communities, Myanmar

**DOI:** 10.3201/eid2706.203223

**Published:** 2021-06

**Authors:** Tierra Smiley Evans, Theingi Win Myat, Nang Sarm Hom, Keersten Michelle Ricks, Min Thein Maw, Zaw Min Oo, Aung Than Toe, Nyein Thu Aung, Pyaephyo Aung, Tin Htun Aung, Paul Kuehnert, Kyaw Zin Thant, Ye Tun Win, Wai Zin Thein, Nicole Rae Gardner, Randal Joseph Schoepp, Christine Kreuder Johnson, Hlaing Myat Thu

**Affiliations:** University of California, Davis, Davis, California, USA (T. Smiley Evans, A.T. Toe, N.T. Aung, N.R. Gardner, C.K. Johnson);; Department of Medical Research, Yangon, Myanmar (T.W. Myat, N.S. Hom, H.M. Thu);; US Army Medical Research Institute of Infectious Diseases, Frederick, Maryland, USA (K.M. Ricks, P. Kuehnert, R.J. Schoepp);; Livestock Breeding and Veterinary Department, Yangon (M.T. Maw, Y.T. Win, W.Z. Thein);; Myanmar Timber Enterprise, Yangon (Z.M. Oo);; Nature Conservation Society Myanmar, Yangon (P. Aung, T.H. Aung);; Myanmar Academy of Medical Science, Yangon (K.Z. Thant)

**Keywords:** Myanmar, zoonoses, communicable diseases, occupational exposure, viruses, tickborne diseases, vector-borne infections, Crimean-Congo hemorrhagic fever virus, Crimean-Congo hemorrhagic fever

## Abstract

Crimean-Congo hemorrhagic fever virus (CCHFV) is endemic in Asia, infecting many animal hosts, but CCHFV has not been reported in Myanmar. We conducted a seroepidemiologic survey of logging communities in Myanmar and found CCHFV exposure was common (9.8%) and exposure to wild animal blood and body fluids was associated with seropositivity.

Crimean-Congo hemorrhagic fever (CCHF), caused by Crimean-Congo hemorrhagic fever virus (CCHFV) (*1*), is a widely distributed arboviral disease. Human CCHF cases have been reported in >30 countries in Africa, the Middle East, Asia, and southeastern Europe (*2*). However, clinical cases or seroprevalence studies for CCHFV have not been reported in Myanmar, likely because active surveillance in humans or animals has not been established (*3*). 

*Hyalomma* ticks, 1 of several CCHFV tick family hosts, are considered the primary vector transmitting CCHFV to humans (*4*). *Hyalomma* tick distribution extends into Myanmar (*5*), and CCHF has been reported in the neighboring countries of China and India (*6*,*7*). Expansion of CCHF from countries with known virus circulation to neighboring countries could occur through introduction of infected ticks, human CCHF cases, or movement of animals (*8*). Climate change also is expected to influence the distribution of *Hyalomma* ticks and CCHFV infections (*9*), increasing the likelihood of disease expansion.

Human CCHFV infections can occur through contact with an infected tick or with blood or tissues from infected humans or animals. People living or working closely with livestock or who have heavy exposure to ticks are at increased risk for CCHFV infection (*10*,*11*). Limited investigations have been performed to identify human exposure to CCHFV caused by wild animal contact, despite serologic evidence for exposure to CCHFV in numerous vertebrate species, including birds (Galliformes and Passeriformes), wild hoof stock (Artiodactyla, Cetartiodactyla, and Perissodactyla), carnivores (Carnivora), bats (Chiroptera), hedgehogs (Erinaceomorpha), rabbits and hares (Lagomorpha), elephants (Proboscidea), rodents (Rodentia), and turtles (Testudinata) (*12*).

CCHF has been designated by the World Health Organization as 1 of 10 high-priority emerging infectious diseases (https://www.who.int/emergencies/diseases/2018prioritization-report.pdf). The designation was based on CCHF’s epidemic and emergence potential, a high case-fatality rate of up to 80% depending on healthcare infrastructure and CCHFV genotype, and a lack of approved medical countermeasures for CCHF (*13**,**14*). Most initial reports of CCHF cases in individual countries have been preceded by epidemiologic surveys that provided evidence of local CCHFV circulation. Our goal was to conduct targeted CCHFV surveillance of Myanmar logging communities, which contain an occupational group with expected high exposure to ticks, domestic livestock, and wild animals.

## The Study

Myanmar uses a traditional method of elephant logging for timber harvest. Consequently, Myanmar has a large network of communities in which loggers live together in temporary villages with their families and occasionally migrant laborers. We collected data from 102 healthy persons from 5 elephant logging communities in and near the Yenwe Forest Reserve, a protected area in central Myanmar, during June 2016–August 2018. Most (57/102) participants, including persons from forest management, logging crews, and elephant caretakers, worked in the protected area and were exposed to forested areas and vectors associated with CCHFV (Table 1). Participants were 17–67 years of age and the median age was 32.5 years. We collected venous blood samples and quantitative medical and behavioral questionnaires from each participant (Appendix).

**Table 1:** Crimean-Congo hemorrhagic fever virus immunoglobulin G seroprevalence by demographic characteristic and occupation among forest logging camp communities, Myanmar

Characteristic	No. positive	No. negative	Period prevalence (95% CI)
Sex			
M	6	56	0.11 (0.05–0.2)
F	4	36	0.11 (0.04–0.23)
Age group, y			
11–20	0	11	0 (0–0.26)
21–30	3	32	0.09 (0.03–0.22)
31–40	5	19	0.21 (0.09–0.40)
41–50	2	13	0.13 (0.04–0.38)
51–60	0	15	0 (0–0.20)
61–70	0	2	0 (0–0.66)
Primary occupation*			
Extractive industries	0	6	0 (0–0.39)
Crop production	0	2	0 (0–0.66)
Livestock farmer	0	1	0 (0–0.79)
Protected area worker, forest ranger	6	51	0.11 (0.05–0.21)
Housewife	1	2	0.33 (0.06–0.79)
Teacher	0	2	0 (0–0.66)
Migrant laborer	0	5	0 (0–0.43)
Hunter	1	7	0.11 (0.02–0.43)
Dependent	3	27	0.09 (0.03–0.24)
Total	10	92	0.11 (0.05–0.17)

*Persons were asked to report their primary occupations but some engaged in additional activities, outside of their primary occupation. For example, persons who did not identify as being a hunter as their primary occupation may have reported hunting.

We used a bead-based MagPix (Luminex Corporation, https://www.luminexcorp.com) assay platform, developed at the US Army Medical Research Institute of Infectious Diseases, to detect specific IgG reactivity against the nucleoprotein of CCHFV. We used molecular detection of conserved regions of the small, medium, and large segments of bunyavirus to detect CCHFV viremia with conventional PCR (Appendix).

We identified previous CCHFV exposure among study participants, but we did not detect any active infections. Study participants did not exhibit any signs of hemorrhagic fever, and none reported having previously suffered symptoms of hemorrhagic-like illnesses. All participants tested negative for bunyaviruses by consensus PCR. Among study participants, 9.8% (10/102) were seropositive for CCHFV by MagPix IgG assay. Samples categorized as positive ranged from 1,124–8,911 mean fold increase (MFI) and a signal-to-noise ratio (S/N) of 33.8–207.8. Negative samples had an MFI of 44–854 and 1–19.9 S/N. Persons 31–40 years of age were significantly more likely to be seropositive for CCHFV (p = 0.05) compared with other age groups. We noted no statistically significant associations between specific occupations and CCHFV exposure (Table 1).

Persons who reported handling live or recently slaughtered primates (age-adjusted odds ratio [OR_age adjusted_] = 5.53; p = 0.020) or wild carnivores (OR_age adjusted_ = 1.3; p = 0.004) in their lifetimes were more likely to have been exposed to CCHFV (Table 2). Handling primates was significantly correlated with handling carnivores (Pearson’s correlation = 0.6; p<0.001). Therefore, we used independent multivariable logistic regression models to adjust for age while assessing the association of CCHFV seropositivity for these 2 factors. More male than female persons reported handling primates (20 male vs. 3 female persons) and carnivores (9 male vs. 3 female persons) and their ages ranged from 19–60 years. Handling primates or carnivores was not statistically significantly associated with any occupational or other behavioral factors.

**Table 2:** Distribution of seropositivity to Crimean-Congo hemorrhagic fever virus among persons exposed to wild and domesticated animals in forest logging camp communities, Myanmar*

Risk factor	Exposed no. persons seropositive/no. tested (%)	Unexposed no. persons seropositive/no. tested (%)	Bivariate model			Multivariable model	
OR	p value		OR	p value
Hunted wildlife							
Ungulate	2/27 (7.4)	8/75 (10.7)	0.67	1.0		NC	NC
Bat	0/1 (0.0)	10/101 (9.9)	2.9†	1.0		NC	NC
Rodent	0/1 (0.0)	10/101 (9.9)	2.9†	1.0		NC	NC
Primate	2/16 (12.5)	8/86 (9.3)	1.39	0.66		NC	NC
Pangolin	2/9 (22.2)	8/93 (8.6)	2.99	0.21		NC	NC
Carnivore	1/9 (11.1)	9/93 (9.7)	1.16	1.0		NC	NC
Any wild animal	4/51 (7.8)	6/51 (11.8)	0.64	0.74		NC	NC
Handled wildlife found dead							
Ungulate	3/32 (9.4)	7/70 (10.0)	0.93	1.0		NC	NC
Bat	1/3 (33.3)	9/99 (9.1)	4.86	0.27		NC	NC
Rodent	1/4 (25.0)	9/98 (9.2)	3.24	0.34		NC	NC
Primate	4/19 (21.1)	6/83 (7.2)	3.37	0.09		NC	NC
Pangolin	1/6 (16.7)	9/96 (9.4)	1.92	0.47		NC	NC
Carnivore	1/10 (10.0)	9/92 (9.8)	1.02	1.0		NC	NC
Any wild animal	8/76 (10.5)	2/26 (7.7)	1.41	1.0		NC	NC
Handled recently slaughtered or live wildlife							
Ungulate	4/26 (15.4)	6/76 (7.9)	2.1	0.27		NC	NC
Bat	1/3 (33.3)	9/99 (9.1	4.86	0.27		NC	NC
Rodent	1/5 (20.0)	9/97 (9.3)	2.42	0.41		NC	NC
Primate	5/23 (21.7)	5/79 (6.3)	4.04	0.04		5.53‡	0.020
Pangolin	2/10 (20.0)	8/92 (8.7)	2.59	0.25		NC	NC
Carnivore	4/12 (33.3)	6/90 (6.7)	6.78	0.02		1.3‡	0.004
Any wild animal	10/88 (11.4)	0/14 (0.0)	3.88†	0.35		NC	NC
Handled live domestic animals							
Goats	0/6 (0.0)	10/96 (10.4)	0.63†	1.0		NC	NC
Pigs	3/23 (13.0)	7/79 (8.9)	1.54	0.69		NC	NC
Poultry	6/57 (10.5)	4/45 (8.9)	1.20	1.0		NC	NC
Cattle	1/8 (12.5)	9/94 (9.6)	1.34	0.58		NC	NC
Elephant	5/43 (11.6)	5/59 (8.5)	1.42	0.74		NC	NC
Any domestic animal	7/60 (11.7)	3/42 (7.1)	1.71	0.52		NC	NC
Slaughtered domestic animals							
Goats	0/0 (0.0)	10/102 (9.8)	NC	NC		NC	NC
Pigs	1/3 (33.3)	9/99 (9.1)	4.86	0.27		NC	NC
Poultry	3/18 (16.7)	7/84 (8.3)	2.18	0.38		NC	NC
Cattle	0/1 (0.0)	10/101 (9.9)	2.9†	1.0		NC	NC
Any domestic animal	9/71 (12.7)	1/31 (3.2)	4.31	0.28		NC	NC

*NC, not calculated; OR, odds ratio.  †Sample odds ratio calculated using unconditional maximum likelihood estimate method.  ‡Evaluated in separate multivariable models, adjusting for age.

Among persons who reported handling wildlife, the highest risk species for CCHFV exposure were primates and carnivores. Although sample size for handling some live or recently slaughtered wild animal taxa were low (for instance, <5 persons each reported handling rodents or bats), we found no statistically significant association between combined wildlife taxa evaluated and CCHFV exposure (p = 1.0; Table 2). 

A bite from an infected tick was not the likely route of exposure to CCHFV in this community. We evaluated occupations associated with increased forest contact, and thus tick habitat, such as resource extraction, protected area worker (forest ranger), or hunter, as a combined variable, but we found no statistically significant association between occupation and CCHFV exposure. 

Contact with domestic animals also was not the likely route of CCHFV exposure in this community. Study participants were not frequently exposed to ruminants, the domestic animal group most reported as associated with CCHFV exposure in endemic countries. Participants were more likely to report contact with pigs or poultry, but these animals have not been identified as amplifying hosts for CCHFV. Contact with live or dead domestic animals of any kind was not associated with CCHFV exposure (Table 2).

Nonhuman primates have not been implicated as natural reservoir hosts or sources of human CCHFV infection. However, rhesus macaques (*Macaca mulatta*) and long-tailed macaques (*M. fascicularis*), which range throughout Myanmar, have been infected with CCHFV in laboratory settings. Rhesus macaques develop viremia without clinical signs, but long-tailed macaques develop signs of clinical illness and viremia similar to disease progression in humans (*15*). Contact with blood or other bodily fluids, including saliva, urine, or feces, during a period of viremia in macaques could lead to human infection. Similarly, wild carnivores have not been implicated as natural reservoir hosts for CCHFV, but red foxes (*Vulpes vulpes*), which are thought to range in Myanmar, and Pallas’s cats (*Otocolobus manul*), which range in central Asia, have demonstrated CCHFV seropositivity and could serve as sources of human infection, particularly through bushmeat hunting, which exposes persons to animal blood and body fluids.

## Conclusions

Our findings indicate that CCHFV is circulating in Myanmar with human infections that are either mildly symptomatic or occurring in populations that fall outside of existing surveillance systems. Although exposure to domestic animal amplifying hosts is the most commonly reported exposure type for human CCHFV infections in endemic countries, our findings show that persons with close contact with wild animal reservoir hosts, especially blood and body fluids of nonhuman primates and carnivores, also are at risk for CCHFV infection. Surveillance of at-risk populations in Myanmar should be expanded to better prepare for potential future outbreaks of CCHF.

Appendix Additional information on Crimean-Congo hemorrhagic fever virus in logging communities, Myanmar.
