# Supplementary material for: Seroepidemiologic Survey of Crimean-Congo Hemorrhagic Fever Virus in Logging Communities, Myanmar
Source: Emerg Infect Dis. 2021 Jun;27(6):1709–13. doi: 10.3201/eid2706.203223 (PMC8153884; doi:10.3201/eid2706.203223)
Supplement: Appendix — Additional information on Crimean-Congo hemorrhagic fever virus in logging communities, Myanmar. [file 20-3223-Techapp-s1.pdf]

Article DOI: https://doi.org/10.3201/eid2706.203223

Seroepidemiologic Survey of Crimean-Congo Hemorrhagic Fever Virus in Logging Communities, Myanmar

Appendix

Project Approvals

Study protocols were reviewed independently and ethical approval was provided by the Institutional Review Board (approval no. 889159–2) and Institutional Animal Care and Use Committee (approval no. 19520) at the University of California, Davis, the Ethics Review Committee of the Department of Medical Research (approval no. 012816), the Forest Department of the Ministry of Natural Resources and Environmental Conservation, the Livestock Breeding and Veterinary Department and the Myanmar Timber Enterprise.

Bead-Based Serologic Assay

Specific immunoglobulin G (IgG) reactivity against Crimean-Congo hemorrhagic fever virus (CCHFV) was detected by using a bead-based assay, MagPix (Luminex Corporation, https://www.luminexcorp.com), developed at the U.S. Army Medical Research Institute of Infectious Diseases. MagPix has demonstrated an enhanced sensitivity profile relative to conventional ELISA (*1*,*2*) and detailed methods have been described previously by Smith et al. (*3*). In brief, recombinant CCHFV nucleoprotein, produced in a baculovirus expression system and based on the IbAr10200 isolate (GenBank accession no. KY484036) as a reference strain, were conjugated to magnetic microspheres by using the xMAP Antibody Coupling Reagent Kit (Luminex Corporation) according to the manufacturer’s instructions. Antigen coupled beads were combined with 1:100 diluted serum and analyzed on the MagPix instrument. Data were evaluated as signal to noise (S/N), with noise being the average median fluorescence intensity of each bead set in response to naive serum samples. We considered any sample with S/N >20 to be seropositive.

Assay Validation

In a comparative study evaluating the immune response to both CCHFV strain Kosova Hoti (GenBank accession nos. DQ133507, EU037902, EU044832) and strain Afg09–2990 (GenBank accession nos. HM452307, HM452306, HM452305) in experimentally infected Cynomolgus macaques (*Macaca fascicularis*), host antibody response was measured from 1–28 days post CCHFV inoculation by viruses derived from both IgG MagPix assay and neutralization assay. Virus-neutralization response was evaluated by using a virus-like particle (VLP) system with glycoproteins based on CCHFV strain IbAr 10200. We observed the emergence of neutralizing antibodies in serum samples by day 9 post infection for both groups, with broadly similar kinetics and endpoint titers between the Hoti and Afg09 infected groups. Mean fold increase (MFI) values averaging 5,737 corresponded with an 80% plaque reduction neutralization titer ($PRNT_{80}$) of 1:100 and MFI values averaging 10,243 corresponded with a $PRNT_{80}$ of 1:400. MagPix and $PRNT_{80}$ values both peaked at day 21 post inoculation.

Determination of Cutoff Value

To determine cutoff values, MFI and S/N were evaluated for a large multiregional serum set, 1,614 samples from Africa and 634 from Asia. Cutoff’s were conservatively set at S/N of 20, far exceeding standard serologic assay cutoff algorithms that would have used 3 standard deviations above the mean of the negative controls.

PCR Assay for Bunyaviral Small, Medium, and Large Segments

Samples were processed for viral detection by using consensus PCR, which enables the universal amplification of sequences from viruses within a given family or genus, and the subsequent discernment of viral strains within. Total nucleic acid was extracted from whole blood by using Direct-zol RNA Miniprep Kits (Zymo Research, https://www.zymoresearch.com) according to the manufacturer’s instructions. Total RNA was reverse transcribed into complementary DNA (cDNA) by using SuperScript III (Invitrogen, https://www.thermofisher.com) according to the manufacturer’s instructions, and 3 assays were used for detection of bunyaviral small, medium, and large segments as described previously by Briese, et al. (*4*).

Statistical Analyses

To evaluate associations between human demographic and animal contact behaviors, all demographic factors, including age, sex, and livelihood, were first evaluated for associations with animal contact behaviors to assess potential confounding. Fisher exact tests were used to determine associations between CCHFV exposure and demographic as well as high-risk human–animal contact behaviors. Odds ratios were calculated by using a conditional maximum likelihood estimate method. For variables, in which 0-count cells were present, we calculated odds ratios by using an unconditional maximum likelihood estimate, Haldane-Anscombe correction. We considered $p<0.05$ statistically significant. Then we used multivariable logistic regression to assess the association between high-risk wild animal contact behaviors and other risk factors that were significant on bivariate analysis. Variables were included when they significantly improved model fit, based on the likelihood ratio test ($p<0.1$), while minimizing the Akaike information criterion. Overall model fit was assessed by using the Hosmer-Lemeshow goodness-of-fit test. All statistical analyses were performed using R version 3.6.1 (R Foundation for Statistical Computing, https://www.r-project.org).

References

1. Satterly NG, Voorhees MA, Ames AD, Schoepp RJ. Comparison of MagPix assays and enzyme-linked immunosorbent assay for detection of hemorrhagic fever viruses. J Clin Microbiol. 2016;55:68–78. PubMed https://doi.org/10.1128/JCM.01693-16

2. Ricks KM, Shoemaker CJ, Dupuy LC, Flusin O, Voorhees MA, Fulmer AN, et al. Development of a bead-based immunoassay using virus-like particles for detection of alphaviral humoral response. J Virol Methods. 2019;270:12–7. PubMed https://doi.org/10.1016/j.jviromet.2019.04.013

3. Smith DR, Shoemaker CJ, Zeng X, Garrison AR, Golden JW, Schellhase CW, et al. Persistent Crimean-Congo hemorrhagic fever virus infection in the testes and within granulomas of non-human primates with latent tuberculosis. PLoS Pathog. 2019;15:e1008050. PubMed https://doi.org/10.1371/journal.ppat.1008050

4. Briese T, Kapoor V, Lipkin WI. Natural M-segment reassortment in Potosi and Main Drain viruses: implications for the evolution of orthobunyaviruses. Arch Virol. 2007;152:2237–47. PubMed https://doi.org/10.1007/s00705-007-1069-z

Human–Animal Contact Behavior Questionnaire

Participant ID: ___________________________
1. Date of Interview _____________________

2. Where are you conducting this interview?
Village/City ______________________________
District __________________________________
Province/State ____________________________
Latitude __________________________ Longitude ___________________________
Interviewer: Please collect GPS coordinates if administering using paper and pen.
Interview/Questionnaire Begins

3. How old are you? __________________________________
If the exact age is unknown, enter the respondent's estimated age.

4. Where do you live?
Village/Town/City ______________________________________
District ____
Province/State ____

Interviewer: Probe for landmarks or nearest known site if area unknown. GPS coordinates to be identified and entered after completion of interview.

5.	How long have you lived there?<br>Select one option.	<1 mo
		1 mo–1 y
		>1–5 y
		>5–10 y
		>10 y

6. How many other people live in the dwelling where you live? ______________________
Skip to question 9 if answer is 0.

7. How many in the dwelling are children less than 5 y old? ______________________

8. How many in the dwelling are male? ___________________

9.	Is the dwelling a permanent structure (that cannot be moved)?	yes
		no

10.	Do you get water from:<br>Select all that apply.	piped in water/water taps
		covered well
		uncovered well/pond/river
		water truck/rainwater harvest
		other

11.	Do you treat your drinking water?	yes
		no

12.	If yes, how do you treat your water?<br>Select all that apply.	boil
		filter
		add chlorine or bleach
		solar disinfection
		other

13.	Is your source for drinking water ever used by animals?	yes
		no

14.	In your dwelling is there a dedicated location for human solid waste/excreta? (e.g., toilet, latrine, designated area)	yes
		no

15.	What is the highest level of education you have completed?<br>Select one option.	primary school
		secondary school
		Finished 10th standard

		college/university/professional
		none

16.	What is the highest level of education that your mother completed?<br>Select one option.	primary school
		secondary school
		Finished 10th standard
		college/university/professional
		none

17.	Since this time last year what are the activities you have done to earn your livelihood?<br>Select all that apply.	1.	extraction of minerals, gas, oil, timber, coal
		2.	crop production
		3.	wildlife restaurant business
		4.	wild/exotic animal trade/market business
		5.	rancher/farmer animal production business
		6.	meat processing, slaughterhouse, abattoir
		7.	zoo/sanctuary animal health care
		8.	protected area worker
		9.	hunter/trapper/fisher
		10.	forager/gatherer/non-timber forest product collector
		11.	migrant laborer
		12.	nurse, doctor, traditional healer, community health worker
		13.	construction
		14.	other:

18. If more than one activity was selected, what is the activity on which you spent the most time since this time last year? *
Write in the activity number from the above list. ___________________

19.	Which best describes your job position?<br>Select one option.	manager (non-government)
		worker (non-government)
		manager (Government)
		Worker (Government)
		live and work at home independently (Skip to question 28)
		Professional (health worker, teacher)
		other:

20. Where do you work? (If different from where you live.)
Village/Town/City ____
District ____
Province/State ____

Interviewer: Probe for landmarks or nearest known site if area unknown. GPS coordinates to be identified and entered after completion of interview.

Medical History Section

In this section, I'm going to ask you about any illness or sickness that is not known or recognized in the community, including by medical or treatment providers.

21.	Where do you usually get treatment for medical problems?<br>Select all that apply.	clinic/health center
		hospital
		mobile clinic
		community health worker
		traditional healer
		dispensary or pharmacy

22.	In your lifetime, have you ***ever*** had an unusual illness with any of the following symptoms (READ ONLY SYMPTOMS)<br>Select all that apply.	fever with headache and severe fatigue or weakness (encephalitis)
		fever with bleeding or bruising not related to injury (hemorrhagic fever)
		fever with cough and shortness of breath or difficulty breathing (SARI)
		fever with muscle aches, cough, or sore throat (ILI)
		fever with diarrhea or vomiting
		fever with rash
		persistent rash or sores on skin
		no (Skip to question 33)
		yes but, none of these symptoms - describe ______________________________

23.	Since this time last year, have you had any of these symptoms?	yes
		no (Skip to question 29)

24.	If yes, which ones?<br>Select all that apply.	fever with headache and severe fatigue or weakness (encephalitis)
		fever with bleeding or bruising not related to injury (hemorrhagic fever)
		fever with cough and shortness of breath or difficulty breathing (SARI)
		fever with muscle aches, cough, or sore throat (ILI)
		fever with diarrhea or vomiting
		fever with rash
		persistent rash or sores on skin
		yes but, none of these symptoms - describe ______________________________

25.	In your opinion, when you were sick, what caused this sickness?<br>Select all that apply.	contact with sick people
		contact with wild animals
		contact with other animals
		bad food or water
		bad spirits/witchcraft
		wound or injury
		I don't know
		other: ______________________

26.	Since this time last year, have any of the people you lived with had any of these symptoms?	yes
		No (skip to question 29)

27.	If yes, which ones?<br>Select all that apply.	fever with headache and severe fatigue or weakness (encephalitis)
		fever with bleeding or bruising not related to injury (hemorrhagic fever)
		fever with cough and shortness of breath or difficulty breathing (SARI)
		fever with muscle aches, cough, or sore throat (ILI)
		fever with diarrhea or vomiting
		fever with rash
		persistent rash or sores on skin
		yes but, none of these symptoms - describe ______________________________

28.	Since this time last year, did anyone you lived with die from this illness?	yes
		no

Movement Section

In this section, I'm going to ask you about any travel you have done since this time last year.

29.	Have you traveled since this time last year?<br>*If answer is no, skip to the next section.*	yes
		no

30. Where have you traveled since this time last year? Anywhere else?
Interviewer: Probe for landmarks or nearest known site if area unknown. GPS coordinates to be identified and entered after completion of interview.
Collect up to 6 locations.

____
____
____
____
____
____

☐

If there are more than six locations check here.
Do not collect additional location information.

31.	Why have you traveled?<br>Select all that apply.	work
		visit family
		moved
		religious reasons

		holiday/vacation
		go to hospital/seek medical care
		go to market
		other: ______________________

Animal Contact Section

In this section, I'm going to ask you about the animals in your life.
If answered "no" under the "in your lifetime" column, then no answer is required under the "Since this time last year" column.

		In your lifetime …	Since this time last year …
32.	Has an animal lived as a pet in or near your dwelling?	yes	yes
		no	no
33.	Have you handled live animals?	yes	yes
		no	no
34.	Have you raised live animals?	yes	yes
		no	no
35.	Have you shared a water source with animals for washing?	yes	yes
		no	no
		don't know	don't know
36.	Have you seen animal feces in or near food before you have eaten it?	yes	yes
		no	no
37.	Have you eaten food after an animal has touched or damaged it?<br>For example, chew marks or scratches	yes	yes
		no	no
		don't know	don't know
38.	Do any animals come inside the dwelling where you live?	yes	yes
		no	no
39.	Have you cooked or handled meat, organs or blood from a recently killed animal?	yes	yes
		no	no
40.	Have you eaten raw or undercooked meat or organs or blood?	yes	yes
		no	no
41.	Have you eaten an animal that you knew was not well /sick?	yes	yes
		no	no
		don't know	don't know
42.	Have you found a dead animal and collected it to eat or share?<br>Select all that apply.	yes	yes
		no	no
43.	Have you found a dead animal and collected it to sell it?	yes	yes
		no	no
44.	Have you been scratched or bitten by an animal?	yes	yes
		no	no

45.	The last time you were scratched, bitten or cut yourself while butchering or slaughtering, what did you do?<br>Select all that apply.	let someone else take over
		wash wound with soap and water
		rinse wound with water
		bandage wound
		visit doctor
		nothing - kept working
		never butcher or slaughter
46.	Do you think there are any risks associated with slaughtering or butchering when you have an open wound?<br>**Interviewer:** Do not read responses.	no
		yes, but I don't know what they are
		yes, it can make you sick
		yes, it can poison you

		yes, it can infect you with a disease
		don’t know
		other

47.	Have you slaughtered an animal?	yes	yes
		no	no

48.	Have you hunted or trapped an animal?	yes	yes
		no	no

		**Circle all headings where “yes” was answered in questions above.**	pet (32)	handled (33)	raised (34)	feces in or near food (36)	in house (38)	cooked/ handled (39)	eaten raw/ under-cooked (40)	eaten sick (41)	found dead (42/43)	scratched/ bitten (44)	slaugh-tered (47)	hunted/ trapped (48)
49.	Ask which animals /mammals for each “yes” category.	Elephant												
		rodents/ shrews												
		bats												
		non-human primates												
		birds												
		carnivores												
		ungulates												
		pangolins												
		Poultry/other fowl												
		goats/ sheep												
		swine												
		cattle/ buffalo												
		dogs												

		cats												

50.	Which crops are at this site?<br>Select all that apply.	coffee, tea, or cocoa plants
		fruit or nut trees
		oil tree plantation
		oil seed crops
		hardwood plantation
		dry grains
		sugar
		vegetable or fruit crops
		pulses/legume
		fiber
		forages
		cover crops
		fallow fields
		rubber
		fruits or nuts

51.	How long have the crops / plantations been growing here?	Less than 1 y
		1−2 y
		2–5 y
		5–10 y
		11–20 y
		21–30 y
		Greater than 30 y

52.	How frequently are crops / plantations harvested?	Less than 1 y
		1−2 y
		2–5 y
		5–10 y
		11–20 y
		21–30 y

53.	What wild animals live in crops / plantations?	rodents/shrews
		bats
		non-human primates
		birds
		carnivores
		ungulates
		pangolins

54.	What type of work or industry is conducted here?<br>Select one option.	underground mining (by shafts or tunnels)
		open surface mining
		hydraulic mining (high pressure water)
		gathering, panning, or collecting
		oil well/gas field
		logging
		other

55.	What product(s) are extracted?<br>Select one option.	coal
		coltan
		diamond or other gemstone
		tin
		gold/silver
		lead
		oil /gas
		timber/plant
		electricity
		other (please specify)

56.	Do you live on the work site?	yes<br>no

57.	To the best of your knowledge, how many people work at this site?	<10<br>10–100<br>101–1000<br>1001–10,000<br>>10,000

58.	How long have you worked at this site?	<1 mo<br>1 mo–1 y<br>>1 y–5 y<br>>5 y

59.	Is there on-site food production?	yes<br>no

60.	If yes, who pays for the cost of growing the food crops?	the company<br>the workers

61.	Is there meat available for consumption?	yes<br>no

62.	If yes, where does the meat come from?<br>Select all that apply.	farmed onsite<br>farmed and purchased from nearby local communities<br>purchased from wholesale market<br>locally caught/hunted<br>bought frozen<br>don't know

63.	Is it possible to consume bushmeat/wild animal meat on or near the site?	yes<br>no

64.	Is there a designated area for rubbish, including animal waste from slaughter/butcher and animal excrement?	yes<br>no

65.	If yes, do people use the designated location for rubbish?	yes<br>no

66.	Do any animals raid food supplies or destroy crops?	yes<br>no

67.	If yes, what animals?<br>Select all that apply.	rodents/shrews<br>bats<br>non-human primates<br>birds<br>carnivores<br>ungulates<br>pangolins<br>poultry/other fowl<br>goats/sheep<br>camels<br>swine<br>cattle/buffalo<br>dogs<br>cats

68.	What is done to stop animals from raiding or destroying food supplies?<br>Select all that apply.	barriers around fields<br>barriers on individual trees<br>fire<br>poison<br>traps<br>shooting<br>loud sounds<br>domestic/guardian animals<br>flooding

		chasing animals out
		nothing

69.	What animals have you hunted since this time last year?<br>Select all that apply.	rodents/shrews
		bats
		non-human primates
		birds
		carnivores
		ungulates
		civits
		pangolins

70.	Since this time last year, what methods have you used to hunt/trap animals?<br>Select all that apply.	snare
		bow
		hands
		gun
		machete
		knife
		net
		cage
		trap
		other

			for consumption at home	for use of animal products at home	for sale for consumption	for sale alive at market	for sale of animal products	live trapping of nuisance animals for trans-location	culling of nuisance animals
71.	What is the purpose of your trapping or hunting? Select all that apply.	rodents/shrews							
		bats							
		non-human primates							
		birds							
		carnivores							
		ungulates							
		pangolins							

Since this time last year, when you hunt or trap:

72.	Are you exposed to blood?	yes
		no
73.	Have you been scratched or bitten?	yes
		no

74.	Since this time last year, have you seen an outbreak of dead wild animals?	yes
		no

75.	If yes, which wild animals?<br>Select all that apply. (add species list)	rodents/shrews
		bats
		non-human primates
		birds
		carnivores
		ungulates
		pangolins

76.	What do you do when you find an animal dead (not in a trap or shot by another hunter)?<br>Select all that apply.	touch it to see if it is still fresh
		butcher in the forest
		smoke or cook in the forest
		take home to prepare
		bury it
		report it to authorities
		take it to sell it
		nothing
		other

77.	How do you transport a dead animal, if you take it?<br>Select all that apply.	not wrapped
		wrapped in leaves or other natural material

		wrapped in plastic
		on yourself / carry by hand
		in a bag
		in a basket

78.	Do you use special protective equipment (e.g., shoes, masks, gloves)?	yes
		no

79.	If yes, which protective equipment?<br>Select all that apply.	shoes/boots
		mask
		clothes
		gloves
		gown/apron

80.	When do you use protective equipment?<br>Select all that apply.	handling animals
		slaughter
		butcher
		always on at work
		other: _____________
